# Global longitudinal strain in cardio-oncology: worth our trouble or more trouble than it’s worth?

**DOI:** 10.1007/s12471-023-01764-5

**Published:** 2023-02-20

**Authors:** Arco J. Teske

**Affiliations:** Department of Cardiology, University Medical Centre Utrecht, Utrecht University, Utrecht, The Netherlands

Trastuzumab is a therapeutic monoclonal antibody specific for the human epidermal growth factor receptor type 2 (HER2), a cell-surface tyrosine kinase receptor overexpressed in approximately 30% of breast cancers. Adding trastuzumab to chemotherapy for patients with early-stage HER2-positive breast cancer reduces the absolute 10-year risk of recurrence by 9% and mortality by 6.5% (relative reduction of approximately a third) [1]. While the benefits are clear, there are concerns regarding this treatment’s potential cardiotoxic side effects. It has been shown that the prevalence of left ventricular (LV) dysfunction in trastuzumab monotherapy is approximately 4%, up to 12% when given sequentially with anthracyclines (further agents with cardiotoxic potential) and even 25% when administered concomitantly with anthracyclines [2]. The prevalence of trastuzumab-induced cardiac dysfunction reported in community-practice-based observation studies is even higher, most likely due to the application of this therapy in an unselected population where pre-existing cardiovascular lifestyle and demographic risk factors are more prevalent. The cardiotoxic effect is unrelated to either dose or duration, meaning that cardiotoxicity can occur at any given time point during treatment. These observations have led to the general recommendation to monitor cardiac function by non-invasive imaging every 3 months [3]. A significant decrease in left ventricular ejection fraction (LVEF) warrants (temporary) discontinuation of trastuzumab therapy, which has been reported in up to 17% in both clinical trials and community practice [2]. It is important to keep in mind that discontinuation of trastuzumab therapy significantly reduces the 5‑year survival from 94% to 80% in early breast cancer patients [4]. Preventing cardiotoxicity by mitigating well-known harmful co-morbidities, such as hypertension, or other preventive (non-)pharmacological interventions would constitute the optimal strategy. Unfortunately, reported trials have generally been negative [5], highlighting the need for other strategies.

This is where the cardio-oncologist could play a pivotal role in optimising anti-cancer treatment. Using echocardiography instead of multigated acquisition scans reduces the individual radiation burden and provides more detailed information beyond the mere LVEF. Secondly, when trastuzumab-induced cardiac dysfunction occurs we can aid the patient in achieving an accelerated recovery by interrupting trastuzumab therapy and promptly initiating heart failure treatment, typically resulting in a complete recovery. Finally, immunotherapy rechallenge with specific cardioprotective strategies in patients with trastuzumab-induced cardiac dysfunction and in those with compromised cardiac function at baseline has recently been shown to be feasible and safe [6]. This multi-disciplinary oncological and cardiac care of patients is vital in improving both short- and long-term cardiovascular and oncological morbidity and mortality.

The recently published 2022 ECS guidelines on cardio-oncology provide specific recommendations regarding risk assessment, cardiovascular monitoring and treatment of this particular cardiotoxicity [3]. Irrespective of the baseline risk, detailed echocardiographic surveillance is still mandatory in all patients every 3 months throughout the entire duration of treatment (typically 1 year). It, therefore, seems appealing to explore other screening modalities that are able to detect cardiotoxicity earlier in its course, allowing earlier interventions aiming to preserve cardiac function and preventing interruption of anti-cancer treatment.

In this issue of the *Netherlands Heart Journal*, van der Linde et al. describe the potential added value of global longitudinal strain (GLS) in detecting trastuzumab-induced cardiac dysfunction in patients with breast cancer and a preserved LV systolic function at baseline [7]. Over 200 echocardiograms performed in 51 patients were available, showing a significant decline in three-dimensional LVEF in 18% of the cohort. Of particular interest is that GLS was found to be a marker indicating dysfunction earlier than our current definition of a decrease in LVEF of 10%. This marker of subclinical LV dysfunction occurred 1 month prior to the drop in LVEF, which seems to be in line with other reports on the added value of GLS. Unfortunately, no baseline patient characteristics could be identified predicting susceptibility to trastuzumab-induced cardiac dysfunction, although it should be noted that the number of patients for such an analysis might be too low to draw such conclusions.

The main question arising from these data is how this will impact clinical decision-making. Should we prescribe cardioprotective drugs based on a decline in GLS? Should we intensify our screening efforts even further in those patients with a GLS decrease? Or are we splitting hairs with over-advanced diagnostics?

Last year, the SUCCOUR trial set out to explore the strategy of initiating heart-failure-directed therapy based on a decline in GLS compared to standard practice where a decline in LVEF dictated intervention. This randomised trial showed no differences between these two strategies regarding either oncological or cardiovascular outcomes, including comparable LVEF values at follow-up [8].

The current study by van der Linde et al. does provide some additional support for the potential GLS holds in detecting cardiotoxicity [7]., However, at the time of writing, there is no evidence indicating that this should change our current healthcare, other than considering intensifying screening in these specific individuals [9]. One way this field will certainly lean is towards the implementation of artificial intelligence and deep learning to predict patients’ risk of developing cardiotoxicity. The availability of routinely collected cardiovascular images (i.e. computed tomography and positron emission tomography scans) and biomarkers in cancer patients, potentially enhanced by an electrocardiogram, could prove to be a valuable asset in determining the baseline susceptibility of cardiotoxicity [10]. This would allow us to better identify at-risk individuals in whom periodic screening would be worthwhile and to safely restrict the use of our services in low-risk individuals.
